# Insight into endophytic microbial diversity in two halophytes and plant beneficial attributes of *Bacillus swezeyi*

**DOI:** 10.3389/fmicb.2024.1447755

**Published:** 2024-08-29

**Authors:** Lei Gao, Jin-Biao Ma, Yin Huang, Murad Muhammad, Hai-Ting Lian, Vyacheslav Shurigin, Dilfuza Egamberdieva, Wen-Jun Li, Li Li

**Affiliations:** ^1^State Key Laboratory of Desert and Oasis Ecology, Key Laboratory of Ecological Safety and Sustainable Development in Arid Lands, Xinjiang Institute of Ecology and Geography, Chinese Academy of Sciences, Urumqi, China; ^2^University of Chinese Academy of Sciences, Beijing, China; ^3^Xinjiang Key Laboratory of Biodiversity Conservation and Application in Arid Lands, Xinjiang Institute of Ecology and Geography, Chinese Academy of Sciences, Urumqi, China; ^4^Faculty of Biology, National University of Uzbekistan, Tashkent, Uzbekistan; ^5^Institute of Fundamental and Applied Research, National Research University TIIAME, Tashkent, Uzbekistan; ^6^State Key Laboratory of Biocontrol, Guangdong Provincial Key Laboratory of Plant Resources, School of Life Sciences, Sun Yat-sen University, Guangzhou, China

**Keywords:** endophytic bacteria, diversity, halophyte, amplicon, *Bacillus* and comparative genomics analysis

## Abstract

This study utilized high-throughput sequencing to investigate endophytic bacteria diversity in halophytic plants *Anabasis truncate* (AT) and *Anabasis eriopoda* (AE) from the Aral Sea region. Following sequence processing, 356 Amplicon Sequence Variants (ASVs) were discovered. The abundance and variety of endophytic bacteria were higher in AT. *Bacillota*, *Pseudomonadota*, *Actinomycetota*, and *Bacteroidota* constituted the dominant in AE, whereas *Pseudomonadota*, *Actinomycetota*, *Bacteroidota*, and *Chloroflexota* constituted the dominant in AT. Biomarkers were identified through LEFSe analysis, showing host-specific patterns. PCoA indicated distinct bacterial community structures. Phylogenetic analysis revealed diverse endophytic bacteria, including potential novel taxa. PICRUSt2 predicted diverse functions for endophytic bacteria in halophytes, indicating recruitment of beneficial bacterial taxa to adapt to extreme hypersaline conditions, including plant growth-promoting, biocontrol, and halophilic/tolerant bacteria. Moreover, the evolutionary relationship, metabolic capabilities, and plant beneficial potentials of the *Bacillus swezeyi* strains, previously isolated from the above two halophytes, were analyzed using comparative genomic and physiological analysis. The *B*. *swezeyi* strains displayed versatile environmental adaptability, as shown by their ability to use a wide range of carbon sources and their salt tolerances. *B*. *swezeyi* possessed a wide range of enzymatic capabilities, including but not limited to proteases, cellulases, and chitinases. Comparative genomic analysis revealed that despite some variations, they shared genetic similarities and metabolic capabilities among the *B. swezeyi* strains. *B*. *swezeyi* strains also displayed outstanding plant-growth-promoting and antagonistic potentials, offering potential solutions to the global food crisis. This study enhances our understanding of microbial diversity in halophytes on saline-alkali land in the West Aral Sea, shedding light on the halophyte microbiome and its collaboration with hosts in highly hypersaline environments. This study also provides a scientific basis for developing high-quality microbial fertilizers and implementing sustainable agricultural practices.

## 1 Introduction

Halophytes, plants that thrive in saline environments, have garnered significant interest due to their unique adaptation mechanisms. Extensive research has highlighted the diverse endophytic bacterial communities associated with these plants, which play crucial roles in promoting plant growth, enhancing stress tolerance, and providing resistance to pathogens (Rampelotto, 2013; Shu and Huang, 2022; Rekadwad et al., 2023). While substantial progress has been made in understanding the functions and diversity of these endophytes, there remain gaps in our knowledge, particularly concerning their roles in specific halophytes and their potential applications.

Recent studies have demonstrated that endophytic bacteria in halophytes contribute to their survival and productivity under extreme saline conditions. However, most research has focused on a limited number of species or regions, leaving a significant gap in our understanding of endophytic communities in less studied halophytes (Shi et al., 2015; Wang et al., 2015; Zhao et al., 2016). Moreover, the functional implications of these microorganisms, particularly in extreme environments like the Aral Sea basin, are not fully explored. Among the halophytes, *Anabasis* L., a representative plant in desert areas worldwide, stands out for its remarkable adaptability to cold, drought, and saline-alkali soils. Its ability to absorb deep soil water makes it ecologically and economically valuable, particularly in arid regions facing adverse environmental conditions (Lauterbach et al., 2019). Therefore, this plant has extremely important ecological and pharmacological value due to their resilience in harsh environments, their value as a source of forage and fuel, and their antioxidant, anti-inflammatory, and antidiabetic properties (Shegebayev et al., 2023; Jiang et al., 2024). *Anabasis* L. is a dominant plant in the Aral Sea region due to its adaptation to high salinity and arid conditions, making it prevalent in these areas (Dimeyeva, 2015). However, there are few papers, articles, and reports about the diversity, function, and application of endophytic bacteria associated with halophytes *Anabasis* L. Thus, it is necessary to explore and isolate the endophytic bacteria diversity and function resources associated with *Anabasis* L.

In this study, we employed high-throughput sequencing to investigate the endophytic bacterial communities in *Anabasis truncata* and *Anabasis eriopoda*, two prominent halophytes in the Aral Sea region. We provide a comprehensive overview of the bacterial diversity and functional traits associated with these plants. We specifically highlight the potential of *Bacillus swezeyi* and its beneficial properties for plant adaptation by strain isolation, genome, and physiological, analysis. This research not only advances our understanding of microbial interactions in extreme environments but also sets the stage for future studies aimed at harnessing these microorganisms for agricultural and ecological applications.

## 2 Materials and methods

### 2.1 Sampling

**Figure 1:**
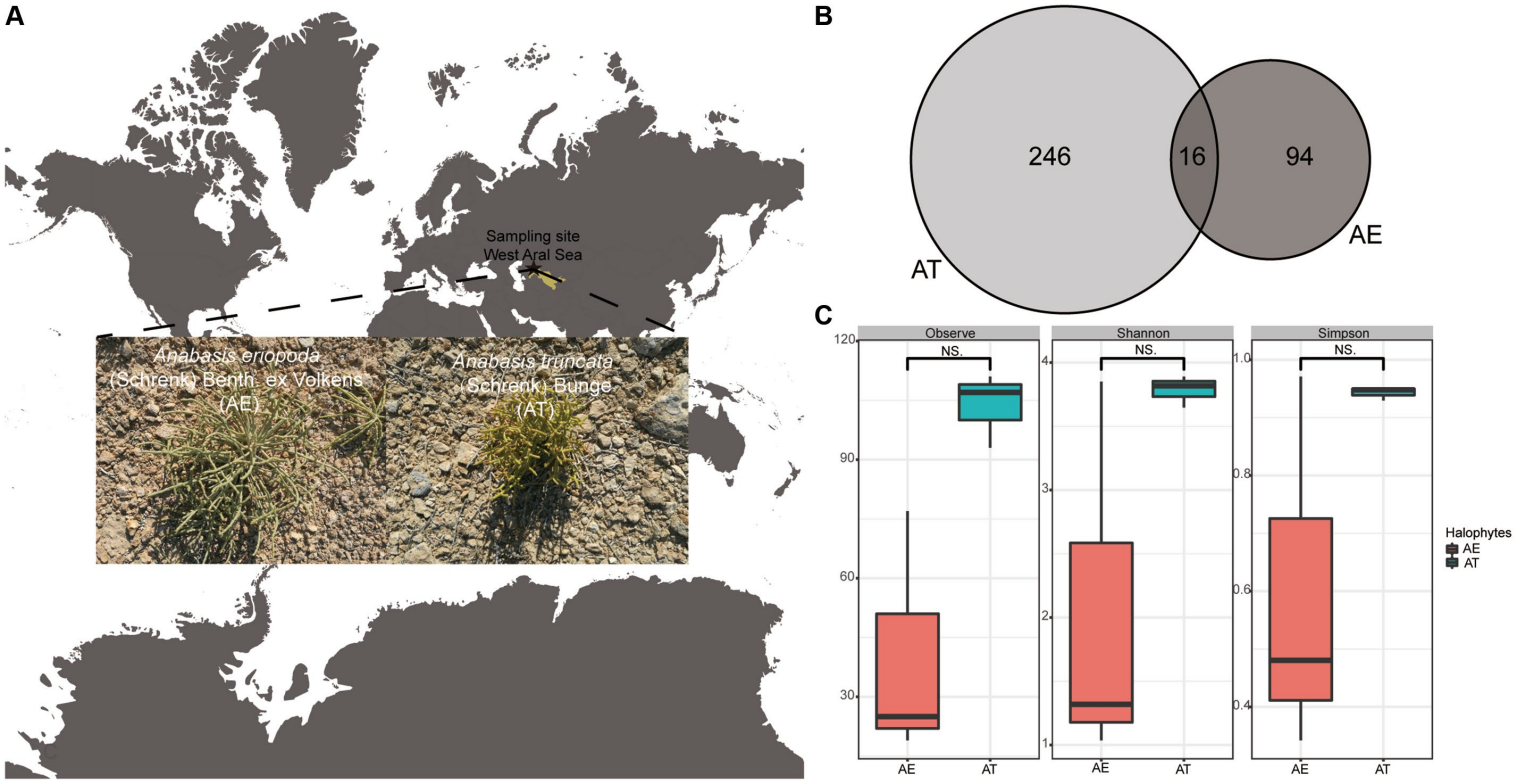
**(A)** Sampling site; **(B)** Venn diagram showing the shared and special ASVs among two different halophytes. **(C)** The boxplots shows the differences of richness and diversity of endophytic bacteria associated with *A. truncata* (Schrenk) Bunge and *A*. *eriopoda* (Schrenk) Benth ex Volkens. Notes: AT, *A. truncata* (Schrenk) Bunge; AE, *A*. *eriopoda* (Schrenk) Benth ex Volkens; NS., non-significant difference.

Two halophyte species were collected from the west Aral Sea (44°,29′, 50.42”N, 58°,12′, 43.80″E), Uzbekistan in August 2018 (Figure 1A). The region is located in Central Asia’s interior, with extremely dry environmental conditions. The Aral Sea region experiences extreme temperature variations, with average temperatures of −12°C (10.4°F) in the north and − 6°C (21°F) in the south during January–February, and 23.3°C (73.9°F) in the north and 26.1°C (79°F) in the south during July, and an annual average rainfall of approximately 100 mm (4 inches)^1^ (Chanjuan et al., 2021). Plant species were collected and identified as *Anabasis truncata* (Schrenk) Bunge and *Anabasis eriopoda* (Schrenk) Benth ex Volkens. Three individual plants of each plant were randomly collected from the same site and labeled AT (*Anabasis truncata*) and AE (*Anabasis eriopoda*), respectively, and placed in aseptic bags, which placed ice immediately and transported to the laboratory of Xinjiang Institute.

### 2.2 Sterilization of plant materials

Three healthy plants (roots, stems, and leaves) were collected and rinsed in water to remove residues. Approximately 10 g of each plant were sterilized with 75% ethanol for 1 min and 5% NaClO (w/v) for 8 min. Sterile deionized water was used for rinsing after disinfection with sodium hypochlorite to ensure the removal of any residual disinfectant. Three sterile plant parts, roots, stems, and leaves were crushed with a sterile mortar and mixed with a phosphate buffer solution (Liu et al., 2017). 100 μL of the last rinse of ddH2O was spread on the TSA plate to check the sterility of plant parts surface sterilization. After 3 days, TSA plates showed no colonial growth, confirming the surface sterilization. In control conditions, the sterile plants were cut into 1–2 cm pieces with a sterile scalpel and dried in the horizontal flow clean bench. Finally, all the sterile plant samples were crushed by a sterile masher and stored at −20°C.

### 2.3 DNA extraction, PCR amplification, and gene clone library construction

The above-prepared samples were sent to MAGIGENE Biotechnology Co., LTD, Guangzhou, China. DNA extraction, PCR amplification, gene clone library construction, and sequencing were completed by MAGIGENE. The target-specific primers 515F (5′- GTGCCAGCMGCCGCGGTAA −3′) and 806R (5′- GGACTACHVGGGTWTCTAAT −3′) were used to amplify the V4 region of the bacterial 16S rRNA gene (Castrillo et al., 2017; Liu et al., 2020; Jiang et al., 2021). After all samples were verified, the Hiseq PE250 platform (Illumina, USA) was sent for high-throughput sequencing.

### 2.4 Amplicon sequence processing and analysis

Primers were trimmed from paired-end raw reads using cutadapt (ver. 4.0) (Kechin et al., 2017). Then, all reads were merged, denoised, filtered chimeras, removed redundancy, and predicted biological sequence using DADA2 (ver. 1.26.0) (Callahan et al., 2016). Each amplicon sequence variant (ASV) was defined as a cluster of reads with 100% sequence similarity. Each ASV was annotated with the Silva database (Release 138.1) using the Q2-feature-classifier plugin (Quast et al., 2012). After that, chloroplasts were deleted from our data using the QIIME2 (ver. 2020.8) (Callahan et al., 2016). The complete endophytic bacterial sequences are available in the NCBI SRA database under accession numbers SAMN17934895 to SAMN17934900.

The dataset without singletons was rarefied to the minimum number of reads recovered from our samples for comparative analysis of species richness (Observed_ASVs, Chao1, se.chao1, ACE, se.ACE) and diversity (Shannon, Simpson, etc.) indices among samples, using the R package “*microeco*” (ver. 1.8.0). One-way ANOVAs and T-tests were used for significant differences in sample alpha diversities. Venn diagrams were produced with the R package *‘VennDiagram’* (ver. 1.7.3). The relationships between endophytic community structures were evaluated by principal coordinate analysis (PCoA). Furthermore, we use the linear discriminant analysis effect size (LEfSe) to identify differentially abundant biomarkers among the two halophytes. The phylogenetic tree was constructed with representative sequences of relatively high abundance (top 100 ASVs) bacteria using Fasttree on the QIIME2 platform and displayed with iTOL (Interactive Tree of Life). Finally, we used PICRUSt2 (Phylogenetic Investigation of Communities by Reconstruction of Unobserved States) to predict the function of endophytic bacteria. The functional prediction of endophytic bacteria based on PICRUSt2 and visualization were completed by Microeco bioinformatics cloud (https://www.bioincloud.tech/) and R (ver. 4.1).

### 2.5 Strains source

The fresh plant samples were surface sterilized in a well-established laboratory according to the procedure described by Liu et al. (2017). And then, endophytic bacteria were acquired by dilution-to-extinction and streak plate methods. The isolation and purified medium was a tryptone soya agar (TSA, Solarbio) containing salt (2% NaCl w/v). The five strains (ES 819, ES 822, ES 82, ES 88, and ES 91) from *Bacillus* genus were identified by the 16S rRNA gene associated with two halophytes *A. truncata* and *A*. *eriopoda,*.

### 2.6 Genome sequencing, phylogenetic, comparative genomics, metabolic potential, and physiological analysis

Five strains underwent DNA extraction using the FastDNA SPIN Kit (MP Biomedical, USA), following the manufacturer’s protocols. At Azenta Life Sciences in Suzhou, China, the whole genomes of five strains were sequenced with Illumina Hiseq X paired-end sequencing. Reads from each dataset were trimmed using Sickle (ver. 1.33) and assembled with SPAdes (ver. 3.15.5) (Joshi Na, 2011; Bankevich et al., 2012). Protein-coding sequences, rRNA, and tRNA were predicted using Prodigal (ver. 2.6.3), tRNA-scan (ver. 2.0.12), and RNAmmer (ver. 1.2), respectively (Lagesen et al., 2007; Chan and Lowe, 2019). The G + C content was calculated from the SPAdes-merged genome file.

A phylogenetic tree of the genome, combined with *Bacillus* genomes, was constructed. Multiple sequence alignments of 120 bacterial marker genes were generated using GTDB-Tk (ver. 2.4.0), and IQ-Tree (ver. 2.3.5) calculated the maximum-likelihood phylogeny (Nguyen et al., 2015; Chaumeil et al., 2019). The comparative analysis analyzed orthologous and exclusive genes between *Bacillus swezeyi* strains using the OrthoFinder program (ver. 2.5.5) within the PGCGAP pipeline (ver. 1.0.35) (Emms and Kelly, 2019; Liu et al., 2021) while ANI was calculated using Pyani (ver. 0.2.12). Functional annotation was performed by querying predicted CDS against the Kyoto Encyclopedia of Genes and Genomes (KEGG) database (Release 108.1) using DIAMOND (ver. 0.7.9; *E*-value <1e^−5^) (Kanehisa and Goto, 2000; Buchfink et al., 2015). Pan-and-core-genome analysis of the *B. swezeyi* genomes was conducted using the PanX program (ver. 1.6.0) (Ding et al., 2017). BGCs were analyzed with antiSMASH (ver. 6.1.1). Plots were generated using R (ver. 4.1) using the ggplot2 package. The predicted CDSs were also uploaded to the KEGG automatic annotation server (KAAS) for annotation by the BLAST program with the BBH (bi-directional best hit) assignment method, and a metabolic pathway map of *B. swezeyi* was generated. Our five *B. swezeyi* strains underwent a comprehensive evaluation activities (Gao et al., 2022). Enzyme production was also quantified with ELISA kits (JINMEI, China) for IAA, ACC deaminase, cellulase, and chitinase.

## 3 Results

### 3.1 Characteristics of sample sequence reads and microbial community richness and diversity

After read-quality filtering using DADA2 and removing chloroplast via QIIME2, 18,275 high-quality clean reads were acquired from two halophytes, ranging from 621 to 7,209 in the samples (Supplementary Table S1). The estimated coverage values (Supplementary Table S2) suggested that the sequencing depths were sufficiently large to capture most of the bacterial diversity in the samples used in this study. A total of 356 amplicon sequence variants (ASVs) were obtained across all libraries at 100% identity. A total of 13 ASVs were shared among two halophytes. The numbers of ASVs exclusive to the AT and AE were 246 and 94, respectively (Figure 1B). Additionally, the richness and diversity of endophytic bacteria related to AT are higher than AE, but these differences are insignificant (Supplementary Table S1; Figure 1C).

### 3.2 Microbial taxonomic analysis

**Figure 2:**
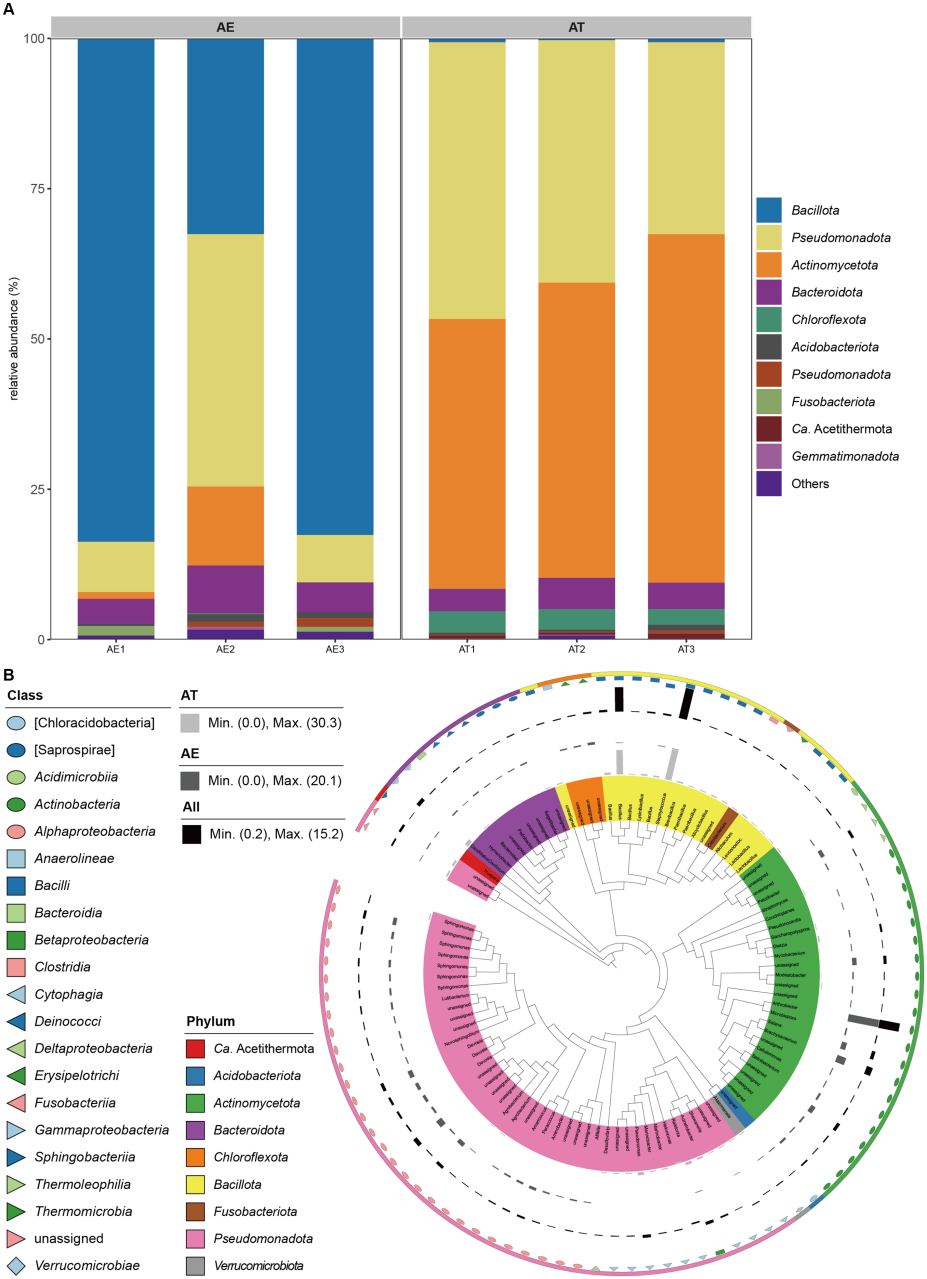
**(A)** Relative abundances of bacteria at the phylum level (top 10) in different samples; **(B)** Taxonomic dendrogram showing top 100 ASVS members of the endophytic bacteria of two different halophytes. The color straps and color ranges identify phyla within the tree. Modules with different shapes and colors represent different classes. Colored bars represent the relative abundance of each ASV in the different halophytes. The taxonomic dendrogram was generated with one representative sequence of each ASV using QIIME2 and displayed with the use of Interactive Tree of Life (iTOL).

High-throughput sequencing revealed the composition of bacterial communities in different samples (Figure 2). The ASVs were classified into 24 phyla, 47 classes, 64 orders, 110 families, and 98 genera (Supplementary Table S1). *Bacillota* (66.31%), *Pseudomonadota* (19.41%), *Actinomycetota* (4.75%), and *Bacteroidota* (5.71%) were the four dominant phyla in the AE. However, *Pseudomonadota* (39.43%), *Actinomycetota* (50.66%), *Bacteroidota* (4.44%), and *Chloroflexota* (3.20%) were the four dominant phyla in the AT (Figure 2A). *Bacilli* (64.37%) and *Gammaproteobacteria* (10.26%) were the two dominant classes in the AE. However, *Actinomycetes* (45.89%) and *Alphaproteobacteria* (36.88%) were the dominant classes in the AT (Supplementary Figure S1). *Bacillales* (63.08%) and *Pseudomonadales* (5.40%) were the dominant orders in the AE. However, *Actinomycetales* (45.89%), *Rhizobiales* (16.09%), and *Sphingomonadales* (11.86%) were the three dominant orders in the AT (Supplementary Figure S2). *Staphylococcaceae* (30.28%) and *Bacillaceae* (27.39%) were the two dominant families in the AE. However, *Micrococcaceae* (20.97%), *Sphingomonadaceae* (8.66%), *Microbacteriaceae* (6.93%), *Hyphomicrobiaceae* (6.82%), and *Dermabacteraceae* (5.96%) were the six dominant families in the AT (Supplementary Figure S3). *Staphylococcus* (30.28%) and *Bacillus* (27.39%) were the two dominant genera in the AE. However, *Arthrobacter* (20.10%), *Sphingomonas* (7.75%), *Devosia* (6.82%), and *Brachybacterium* (5.90%) were the four dominant genera in the AT (Supplementary Figure S4). The annotation findings revealed additional unassigned taxa at the family and genus levels. The barplot indicated that the remaining taxa had a higher relative abundance in the annotation results at these levels. High-throughput sequencing revealed distinct bacterial communities in AE and AT, with *Bacillota*, *Pseudomonadota*, and *Actinomycetota* predominating in AE, while *Pseudomonadota*, and *Actinomycetota*, were dominant in AT, showing significant variations in class, order, and family distributions, along with unassigned taxa at family and genus levels.

In Figure 2B, the representative sequences of this study’s top 100 abundant endophytic bacteria were selected to construct the phylogenetic tree. Based on the phylogenetic relationship, it can be found that the endophytic bacteria of the two halophytes have the characteristic of high diversity. The top 100 abundant endophytic bacteria belonged to 9 phyla, 21 classes, 30 orders, 57 families, and 48 genera. Among these, 43 ASVs belonged to *Pseudomonadota*, (25) *Actinomycetota*, (16) *Bacillota*, (9) *Bacteroidota*, (3) *Chloroflexota*, (1) *Ca.* Acetithermota, (1) *Acidobacteriota*, (1) *Fusobacteriota*, and 1 to *Verrucomicrobiota*. The genera *Bacillus* and *Staphylococcus* affiliated to the class *Bacilli* of the phylum *Bacillota* were enriched in AT, while the genus *Arthrobacter* affiliated to the class *Actinomycetes* of the phylum *Actinomycetota* was enriched in AE. Besides, the phylogenetic tree showed that the top 100 abundant endophytic bacteria associated with the two halophytes contained some potential novel unassigned taxa.

### 3.3 Biomarkers analysis of endophytic bacteria between two halophytes

**Figure 3:**
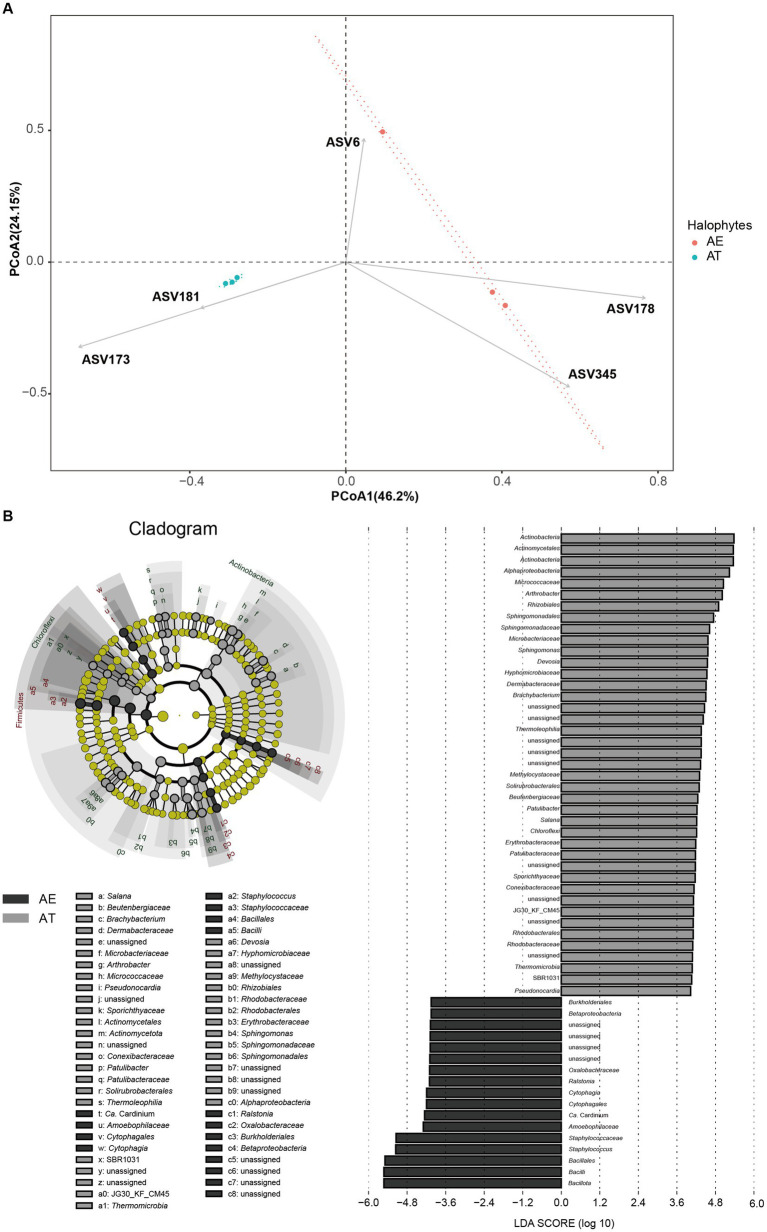
**(A)** Endophytic bacteria with relative abundance differences based on LEFSe analysis; **(B)** Principal coordinate analysis (PCoA) of different microbiota in different samples based on the unweighted-unifrac distance.

Beta-diversity analysis based on PCoA of the unweighted-unifrac distance was performed to compare the microbial compositions of different samples. PCoA revealed the samples’ main variations in bacterial community composition (Figure 3A). The highest variations in the endophytic bacteria of different samples were 46.2% (PCoA1) and 24.15% (PCoA2), representing a strong separation between the two halophytes. The samples from the same halophyte were predominantly grouped, whereas others exhibited a moderately distinct separation between two halophytes. Figure 3A further shows that ASV173 (*Arthrobacter*) and ASV181 (*Brachybacterium*) have a high AT enrichment, whereas ASV6 (*Pseudomonas balearica*), ASV178 (*Staphylococcus*), and ASV345 (*Bacillus*) have a high AE enrichment.

Significant differences were observed in the compositions of endophytic bacterial and fungal communities between two halophytes. The results of the LEFSe analysis showed significantly different taxa abundances among the two halophytes. Between the two halophytes, 58 biomarkers were found, with 41 biomarkers substantially enriched in at and 17 biomarkers considerably enriched in AE (LDA > 4, *p* < 0.05, Figure 3B). Among all biomarkers (116), 41 enriched biomarkers in AT mainly were *Actinomycetota*, *Actinomycetales*, *Alphaproteobacteria*, *Micrococcaceae*, *Arthrobacter*, *Rhizobiales*, *Sphingomonadales*, *Sphingomonadaceae*, *Microbacteriaceae*, *Sphingomonas*, etc., which were all affiliated to three phyla (*Actinomycetota*, *Pseudomonadota*, and *Chloroflexota*). While 17 enriched biomarkers in AE mainly included *Burkholderiales*, *Betaproteobacteria*, *Oxalobacteraceae*, *Ralstonia*, *Cytophagia*, *Cytophagales*, *Staphylococcaceae*, *Staphylococcus*, *Bacillales*, *Bacilli*, etc., which were all affiliated to three phyla (*Actinomycetota*, *Pseudomonadota*, and *Bacteroidota*).

### 3.4 Microbial insights about halophytes adapting to the extreme hypersaline environment

**Figure 4:**
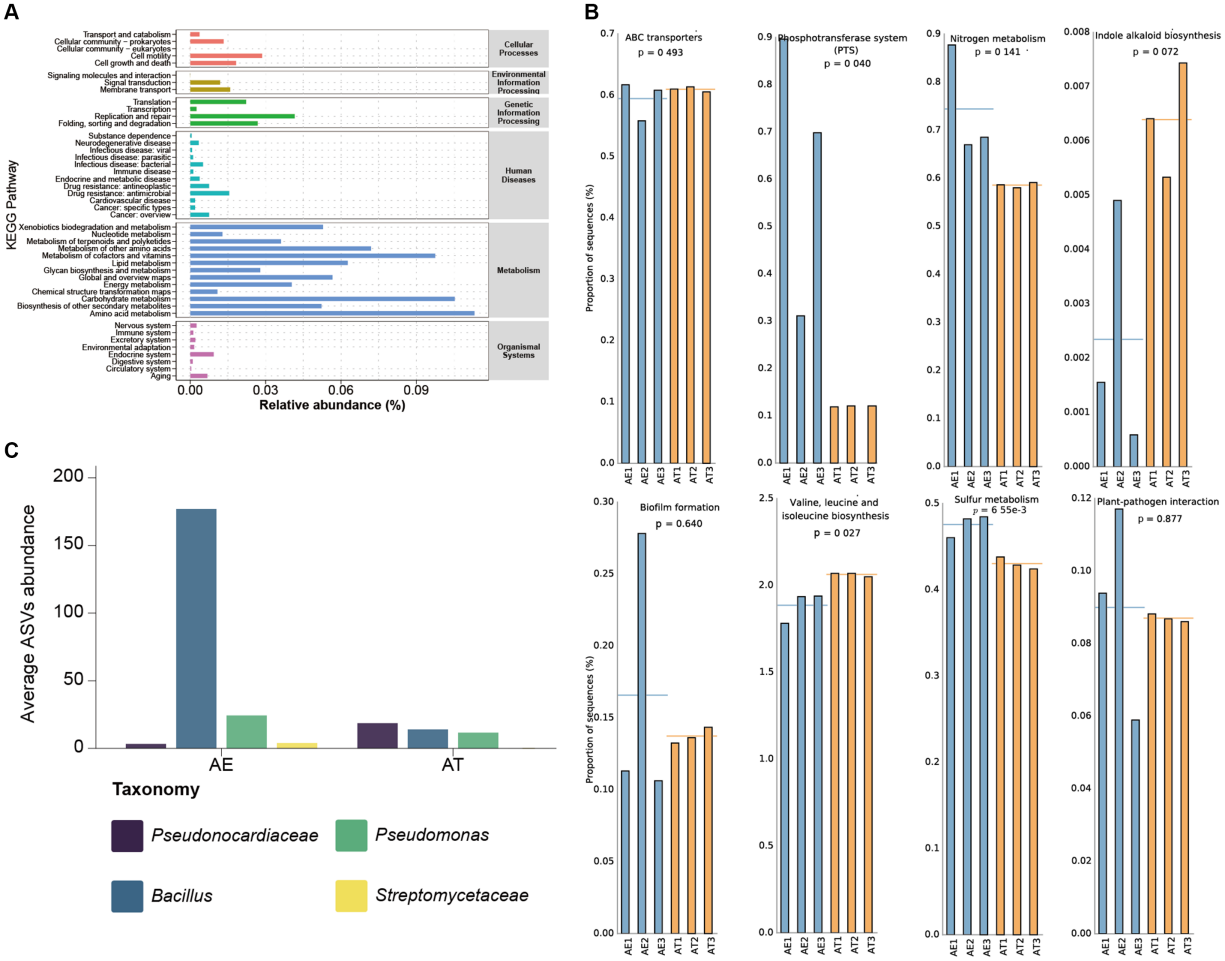
**(A)** The abundance distribution map of microbial function based on KEGG prediction; **(B)** Pathway abundance related to hypersaline environment adaptivity and plant beneficial traits; **(C)** Plant beneficial bacterial taxa’s ASVs abundance.

Considering the overall context of the calculated metagenomes, we also investigated the role of the microbiota by employing the PICRUSt2 algorithm. Through function prediction of endophytic bacteria based on the green genes database, 45 relevant KEGG categories were obtained, including cellular processes, environmental information processing, genetic information processing, human diseases, metabolism, and organismal systems. We selected level 2 of the KEGG predicting pathway to draw the abundance distribution map of microbial function (Figure 4A). The KEGG pathways connected to metabolism, such as Amino acid metabolism, Carbohydrate metabolism, Metabolism of cofactors and vitamins, Metabolism of other amino acids, Lipid metabolism, Biosynthesis of other secondary metabolites, etc., are all characterized by high abundance. Furthermore, the KEGG pathways related to Genetic Information Processing, Cellular Processes, and Environmental Information Processing also exhibited moderate abundance.

Our PICRUSt2 prediction results show some metabolic pathways with plant-beneficial traits (Figure 4B). High abundances of ABC transporter- and phosphotransferase system (PTS) related sequences were predicted in all samples, which may assist the host plants in adapting to extremely hypersaline environments. Additionally, there are relative high abundances of plant-beneficial metabolism, such as nitrogen metabolism, indole alkaloid biosynthesis, biofilm formation, and BCAAs (Branched-chain amino acid) biosynthesis. Plant growth is inseparable from the supplement of nitrogen sources. The nitrogen and sulfur metabolism process of microorganisms, especially nitrogen fixation, provides sufficient nitrogen and sulfur sources for the growth of host plants. Plant hormones are crucial for the growth and development of plants, particularly during the initial stages and the development of plant tissues. It can potentially impact the elongation and division of plant cells, the growth of primary lateral roots and hypocotyls, the development of vascular tissue, the establishment of plant geotropism and phototropism, and the formation of root hairs and flower organs. The synergy between plant-beneficial bacteria is considered to function through biofilm formation. Biofilm is an important medium for plant growth-promoting rhizobacteria (PGPR) colonization and key nutrient exchange. BCAAs refer to the three common amino acids, leucine, valine, and isoleucine, which can promote anabolism to adapt and colonize in different plant ecological niches. The predicted relative high abundance of plant-pathogen interaction pathways indicated that some plant biocontrol bacteria may be colonized in different halophytic ecological niches to protect halophytes against phytopathogens.

Through the in-depth mining of our sequencing data, it was found that there are some plant-beneficial taxa in the endophytic community of halophytes, such as *Pseudonocardiaceae*, *Bacillus*, *Pseudomonas*, *Streptomycetaceae*, etc. (Figure 4C). *Pseudonocardiaceae* is a group of highly stress-tolerant *Actinomycetes*, which may alleviate salt stress and help halophytes adapt to extremely hypersaline environments (Hamedi et al., 2013). At present, the relevant research on plant microbiomes shows that *Bacillus* and *Pseudomonas* are the main plants’ beneficial taxa, especially in all kinds of plants beneficial *Bacillus* is the main microbial fertilizer production strain (Kour et al., 2020; Dasgupta et al., 2021). *Bacillus* and *Streptomycetaceae* are the main plant biocontrol taxa to protect against pathogen infection (Glaeser and Kämpfer, 2016; Otani et al., 2022; Khan et al., 2023).

### 3.5 Genome characteristics, phylogenetic and comparative genome analysis

**Figure 5:**
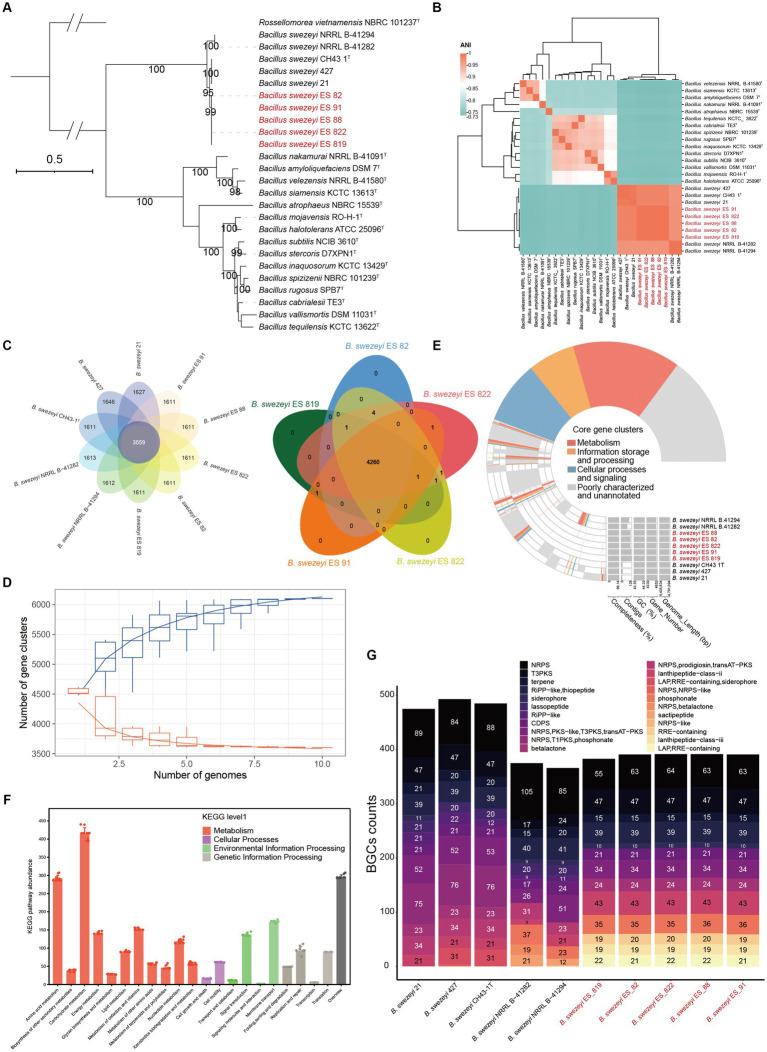
**(A)** Phylogenomic tree of species of the genus *Bacillus*, Bootstrap values (>90) based on 1,000 resamplings are marked gray points at the nodes. *Rossellomorea vietnamensis* NBRC 101237^T^ was used as an outgroup. ModelFinder used the Akaike Information Criterion (AIC), Corrected Akaike Information Criterion (Correct AIC), and Bayesian Information Criterion (BIC) to select the best-fit model (mtInv+I + R3); **(B)** ANI value shared among the related *Bacillus* species; **(C)** The flower and venn diagram illustrated the number of unique and shared protein-coding genes between *B*. *swezeyi* strains; **(D)** Pan- and core-genome evolution and functional annotations between *B*. *swezeyi* strains; **(E)** The pan-genome profile of *B*. *swezeyi* strains. **(F)** KEGG pathway abundance of *B*. *swezeyi* strains; **(G)** Biosynthetic gene clusters of *B*. *swezeyi* strains.

The halophytes *A. eriopoda* and *A. truncata* were used to isolate five strains of endophytic Bacillus bacteria, typically regarded as an important class of plant-beneficial microbes. The general genomic features of *B*. *swezeyi* are summarized in Supplementary Table S3. The genome size of *B*. *swezeyi* strains ranges from 4,420,959 to 4,791,094 bp bases with 43.55–44.33% GC content. The phylogenomic tree (Figure 5A) based on the genomes of five *B*. *swezeyi* strains and its related *Bacillus* taxa supported the closer phylogenetic relationship with *B*. *swezeyi* CH43-1^T^. Average nucleotide identity (ANI) value showed high genomic similarities between *B*. *swezeyi* genomes (Figure 5B). Among all calculated strains, ANI value between our five *B*. *swezeyi* strains and *B*. *swezeyi* CH43-1^T^ were higher than the cut-off points for delineation of genomic species, indicating they belong to different strains in the same species. Comparative genomics analysis based on orthologs among the *B*. *swezeyi* genomes retrieved using Orthofinder showed that our five *B*. *swezeyi* strains shared 3,559 core proteins with other *B*. *swezeyi* strains.

In contrast, more than 1,000 proteins were exclusive for *B*. *swezeyi* strains (Figure 5C). This means a great difference exists between our five *B*. *swezeyi* strain and the current *B*. *swezeyi* strain in the database. The extent of genetic variation among *B*. *swezeyi* strains was evaluated by analyzing the distribution of conserved (core) and species-specific (unique) genes using a pan-genome analysis. A limited number of core gene clusters, involved in various basic biological processes such as metabolism, information storage and processing, cellular processes, and signaling, were identified among these genomes.

Nevertheless, numerous strain-specific genes involved in metabolism, information storage and processing, cellular processes and signaling, and uncharacterized and poorly annotated genes, were present, contributing to the unique metabolic functions of each strain of this genus (Figures 5D,E). The properties and statistics of genes associated with the KEGG functional pathways are displayed in Figure 5F. The most abundant KEGG functional pathways in the *B*. *swezeyi* genomes were assigned to metabolism, including carbohydrate metabolism, amino acid metabolism, overview, etc. Notably, for an overall comparison among the genomes of *B*. *swezeyi* strains, all the genomes exhibited a difference in gene number in some KEGG functional pathways categories to some extent, suggesting that *B*. *swezeyi* genomes have also been characterized by variabilities. In addition, we also utilized antiSMASH to make predictions on the BGCs of related strains, which uncovered the similarities and differences between the strains’ secondary metabolites. All strains showed certain differences in the type and abundance of BGCs. However, some BGCs showed universality among most strains. The BGCs were mainly divided into 22 categories, including NRPS, PKS-like, terpene, transAT-PKS, NRPS, T3PKS, transAT-PKS, NRPS, betalactone, transAT-PKS, T3PKS, NRPS, RiPP-like; LAP, RRE-containing; and other (Figure 5G).

### 3.6 Construction of metabolic pathway and physiological verification of *Bacillus swezeyi*

**Figure 6:**
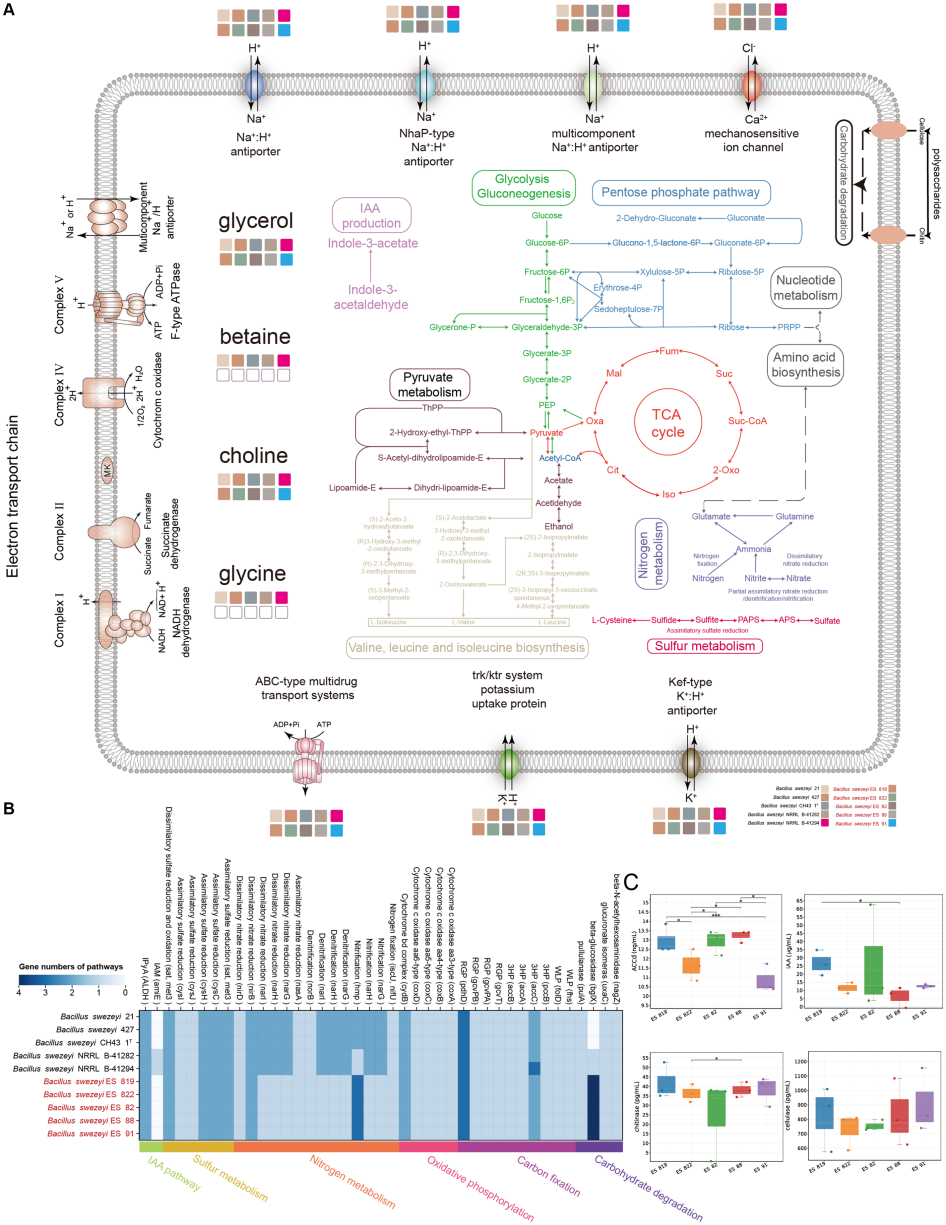
**(A)** Overview of metabolic potentials in *B*. *swezeyi*, including glycolysis, gluconeogenesis, the pentose phosphate pathway, pyruvate metabolism, tricarboxylic acid (TCA) cycle, nitrogen and sulfur metabolism, membrane transporters, potential mechanisms for regulating the intracellular pH and cytoplasmic ion content, etc. This figure was generated according to the gene data in Supplementary Table S4 using Adobe Illustrator 2022 software. **(B)** KEGG pathways of *B*. *swezeyi*, categories of pathways are represented at the bottom side of the heatmap by different colors, the color of each cell refers to the gene numbers involved in each pathway, Corresponding genes involved in each pathway are shown in parentheses; **(C)** ACCD production, IAA, cellulase, and chitinase species of the five *B*. *swezeyi* strains. PEP, phosphoenolpyruvate; PRPP, phosphoribosyl pyrophosphate; Oxa, oxaloacetate; Mal, malate; Fum, fumarate; Suc, succinate; Suc-CoA, succinate-CoA; Oxo, oxoglutarate; Iso, isocitrate; Cit, citrate; The method employed for the differential comparison was the *t*-test. * refers to *p* < 0.05; ** refers to *p* < 0.01; *** refers to *p* < 0.001.

Genome annotations and physiological experiments revealed that *B*. *swezeyi* participated in diverse metabolisms such as carbon, nitrogen, sulfur, phosphorus, etc. To learn the physiology of *B. swezeyi* globally, we rebuilt the metabolic pathways and cellular transport systems (Figure 6A). *B. swezeyi* strains encoded the glycolysis (Embden-Meyerhof) pathway, the pentose phosphate pathway, and pyruvate metabolism. In addition, *B*. *swezeyi* possessed crucial genes involved in carbohydrate degradation pathways, such as *nagZ*, *uxaC*, *bglX*, and *pulA*. In a nutshell, *B*. *swezeyi* strains played an important role in the carbon cycle and participated in key carbon cycle processes such as organic carbon oxidation and fertilization (Figure 6A). In terms of energy metabolism, the genome-encoded oxidative phosphorylation pathway, including a cytochrome b6-f complex subunit (*petC*), cytochrome c oxidase (*ctaCDE*), and F-type ATPase (*atpABCDEFGH*). The synergy between plant-beneficial bacteria was considered to function through biofilm formation. Biofilm is an important medium for plant growth-promoting rhizobacteria (PGPR) colonization and key nutrient exchange. In *B*. *swezeyi* genomes, we also detected some genes related to biofilm formation (Supplementary Table S4). BCAAs (Branched-chain amino acids) refer to the three common amino acids: leucine, valine, and isoleucine. These could promote some plants’ beneficial bacteria anabolism to adapt and colonize in different plant ecological niches. We also detected a completed pathway of BCAAs biosynthesis in the *B*. *swezeyi* genomes (Figure 6A). From the metabolic level of genome annotation, we revealed that *B*. *swezeyi* strains have certain potential plant-beneficial activities. For excavating into the salt tolerance potential of *B*. *swezeyi*, these *B*. *swezeyi* genomes could encode many function genes for the compatible solute, Na^+^:H^+^ antiporter, Trk-type K^+^ uptake transporters, Kef-type antiporter, and other salt-stress resistance genes (Figure 6A; Supplementary Table S5). In IAA (indole-3-acetic acid) biosynthesis pathways, the IPyA pathway was the major IAA biosynthetic pathway in these *B*. *swezeyi* strains. This indicates that *B*. *swezeyi* is a group of salt-tolerant, disease-resistant, and growth-promoting functional microorganisms, which has the potential to contribute to the improvement of saline-alkali soil for agricultural use. Two carbon fixation pathways genes were found in *B*. *swezeyi* strains, including the 3-hydroxypropionate bicycle (3HP-bicycle) and the Reductive Glycine Pathway (RGP) (Figure 6B). Carbon fixation by microorganisms increases soil organic matter, provides nutrients, and improves soil structure, which is beneficial for plant growth. In addition, it indicated that *B*. *swezeyi* also probably participates in certain key steps of nitrogen and sulfur metabolism, such as nitrogen fixation (*nifU*), nitrification, assimilatory nitrate reduction, assimilatory sulfate reduction (Figure 6B; Supplementary Table S6). These processes collectively enhance essential nitrogen and sulfur for proteins and defense in plants, crucial for constructing cells, proteins, and nucleic acids, thereby emphasizing their pivotal roles in supporting plant development. Additionally, *B*. *swezeyi* strains included genes that coded for organic phosphorus mineralization, polyphosphate degradation, polyphosphate synthesis, phosphorus transporter, and regulatory such as *pstA*, *pstB*, *pstC*, *ugpQ*, *ppaC*, *pstS*, *phoA*, *pit*, *PK*, *ppnK*, *ndk*, *phoD*, *phoR* (Supplementary Table S4).

According to the results of Biolog Gen III, *B*. *swezeyi* could utilize dextrin, D-maltose, D-cellobiose, gentiobiose, sucrose, D-raffinose, α-D-lactose, D-melibiose, β-methyl-D-glucoside, D-salicin, N-acetyl-D-glucosamine, N-acetyl-β-D-mannosamine, L-fucose, L-rhamnose, inosine, 1% sodium lactate, D-serine, D-mannitol, myo-inositol, glycerol, D-aspartic acid, L-pyroglutamic acid, L-serine, guanidine HCl, pectin, L-galactonic acid lactone, D-gluconic acid, D-saccharic acid, methyl-pyruvate, α-keto-glutaric acid, D-malic acid, L-malic acid, bromo-succinic acid, lithium chloride, tween 40, γ-amino-butryricAcid, α-hydroxy-butyric acid, α-keto-butyric acid, acetoacetic acid, propionic acid, acetic acid, formic acid, aztreonam, sodium butyrate. These results demonstrated the metabolic versatility of these strains. The API 20NE experiments showed that *B*. *swezeyi* was found to have many abilities, including nitrate reduction, glucose fermentation, arginine dihydrolase, urease, β-glucosidase, hydrolyse gelatin, β-galactosidase, glucose, arabinose, mannose, mannitol, N-acetyl-β-glucosamine, maltose, malic acid, and trisodium citrate assimilation. According to the results of experiments conducted by API ZYM, these strains shared the following enzyme activities: alkaline phosphatase, (C4) esterase, and naphthol-AS-BI-phosphohydrolase (Supplementary Table S7). *B*. *swezey* strains exhibited ACC deaminase-produceing properties, as shown by the result of the physiological experiment (Figure 6C). ACC is one of the precursors involved in ethylene synthesis in plants. The enzyme ACC deaminase degrades ACC into ammonia and α-keto butyric acid, lowering the ethylene levels in plants and promoting plant growth (Glick et al., 2007). Analysis of the microbial indoleacetic acid (IAA) with ELISA kit showed that *B*. *swezey* strains could produce IAA (Figure 6C), suggesting that these strains could promote plant growth. Microbial Chitinase Detection Kit analysis shows that *B*. *swezey* strains could produce chitinase (Figure 6C). *B*. *swezey* strains producing chitinase can aid in protecting against plant diseases by breaking down the cell wall components (chitin) of plant pathogenic fungi (Kumar et al., 2018). When analyzed with the Microbial Cellulase Detection Kit, five *B*. *swezey* strains showed the ability to produce cellulase (Figure 6C). The results above indicate that *B*. *swezey* may belong to plant probiotics. Further in-plant testing is essential to validate this potential in future research.

## 4 Discussion

Endophytes, including bacteria, fungi, and actinomycetes, are critical for enhancing plant growth, disease resistance, and stress tolerance, which is particularly relevant for halophytes enduring saline-alkali conditions (Wani et al., 2015; Afzal et al., 2019). These mutualistic microorganisms reside within plant tissues, benefiting both the host and themselves without causing harm (Kushwaha et al., 2020; Rana et al., 2020). The diversity and composition of endophytic communities are influenced by plant species, genotypes, and environmental conditions (Wei and Ashman, 2018). Our study investigated endophytic bacterial diversity in halophytes *A. truncata* (Schrenk) Bunge and *A*. *eriopoda* (Schrenk) Benth ex Volkens grown on the west Aral Sea. Based on diversity analysis, the endophytic bacterial richness and diversity were higher in *A. truncata* (Schrenk) Bunge than in the *A*. *eriopoda* (Schrenk) Benth ex Volkens, at non-significant level (Figure 1C).

Community composition analysis showed that *Pseudomonadota*, *Bacteroidota*, and *Actinomycetota* were core species in the halophytic endosphere (Figure 2A), as same with other studies (Pei et al., 2017; Szymańska et al., 2018; Huang, 2019; Li et al., 2020). Moreover, *Bacillota*, *Pseudomonadota*, *Actinomycetota*, and *Bacteroidota* were the four dominant phyla in the AE, while *Pseudomonadota*, *Actinomycetota*, *Bacteroidota*, and *Chloroflexota* were the four dominant phyla in the AT. These findings align with various studies on endophytic bacterial communities in similar environments. Akinsanya et al. found *Pseudomonadota*, *Bacillota*, *Actinomycetota*, and *Bacteriodetes* as the dominant phyla in the root using high-throughput sequencing technology to study the diversity of endophytic bacteria in the medicinal halophyte *Aloe vera* (Akinsanya et al., 2015). Zhao et al. evaluated the diversity and potential growth-promoting effect of the endophytic bacterial community of halophyte *Salicornia europaea* L. by combining high-throughput sequencing and culture-dependent methods. The results showed that the endophytic bacterial community was dominated by *Pseudomonadota*, followed by *Bacteroidota*, *Actinomycetota*, and *Bacillota*. In the study of endophytic bacteria in saline-alkali tolerant rice seeds, the high-throughput sequencing technology based on the Illumina Miseq platform was used to reveal the “core microbiota.” The results showed the dominant phylum was *Pseudomonadota* which represents the core microbiota in saline-alkali-tolerant rice seeds (Wang et al., 2021). Tian and Zhang concluded that the 37 phyla were identified in an Illumina-based analysis of endophytic and rhizosphere bacterial diversity of the coastal halophyte *Messerschmidia sibirica*, with *Pseudomonadota* and *Actinomycetota* being the dominant phyla (Tian and Zhang, 2017). Our previous study used the culture-dependent method to investigate the diversity and antifungal activity of endophytic bacteria associated with halophytes grown on the shore of the western Aral Sea in Uzbekistan. Finally, we isolated and identified 289 endophytic bacterial strains belonging to three common bacterial phyla: *Bacillota*, *Actinomycetota*, and *Pseudomonadota*, from the nine halophytic samples (Gao et al., 2021). The study related to the diversity of endophytic bacteria in the the medicinal halophyte *Glehnia littoralis* based on Illumina sequencing demonstrated that the *Actinomycetota* and *Pseudomonadota* were dominant in all the samples at the phylum level. Singh et al. studied the biodiversity of endophytic bacterial from halophyte *Salicornia brachiata,* and they obtained 336 strains belonging to three major taxa: *Bacillota*, *Pseudomonadota*, and *Actinomycetota* (Singh et al., 2021). Our research group’s previous studies on the diversity and growth-promoting activity of endophytic bacteria of halophyte *Lycium ruthenicum* Murr showed that a total of 109 endophytic bacteria affiliated to 3 phyla were isolated using nine different selective media, in which *Actinomycetota* was also the dominant taxon (Liu et al., 2019). Szymaska et al. discovered that *Pseudomonadota* and *Bacteriodetes* were the dominant endophytic bacterial phyla related to halophyte *Salicornia europaea* at two different salinity sites using the Miseq Illumina sequencing approach. Additionally, the relatively high abundances of *Planctomycetes* and *Acidobacteria* were detected in this halophyte grown on high saline sites, while the relatively high abundances of *Bacillota*, *Actinomycetota*, and *Chloroflexota* were detected in this halophyte grown on low saline sites (Szymańska et al., 2018). By above study, it was made clear that the dominant phyla of endophytic bacteria are depemedt on soil salinity level.

The results of LEFSe indicated some biomarkers were common between two halophytes (Figure 3A). The result of the PCoA analysis showed some differences in the community structure of endophytic bacteria between *A. truncata* (Schrenk) Bunge and *A*. *eriopoda* (Schrenk) Benth ex Volkens (Figure 3B). However, to further determine the cause of this difference, we need further comprehensive sampling (including recording plant age, growth state, and soil physical and chemical properties) for sequencing verification. Phylogenetic analysis revealed that there is a few unassigned endophytic bacteria in halophytes *A. truncata* (Schrenk) Bunge and *A*. *eriopoda* (Schrenk) Benth ex Volkens (Figure 2B). Meanwhile, the phylogenetic tree constructed using ASV of the top 100 abundance showed the characteristics of high diversity (Figure 2B). In the future, one of our research works is exploring a culture-dependent method that will be used to isolate potential novel endophytic bacteria with plant growth-promoting and stress resistance function.

The predicting abundance of KEGG pathways for endophytic bacteria mainly focused on metabolism, such as amino acid metabolism, carbohydrate metabolism, and metabolization of cofactors and vitamins (Figure 4A). In our PICRUSt2 prediction results, there are some metabolic pathways with plant-beneficial traits to adapt to extremely hypersaline environments, such as ABC transporter and phosphotransferase system (PTS), nitrogen metabolism, indole alkaloid biosynthesis, biofilm formation (Li et al., 2024), and BCAAs (Branched-chain amino acid) biosynthesis (Figure 4B) similarly results were as described by (Sun et al., 2022). It is also necessary to further study the metabolic diversity of endophytic bacteria, which can provide new insights into the mutually beneficial symbiosis between endophytic bacteria and host plants. The comparative genomic study of *B*. *swezey* reveals its significant ecological impact and shows diverse functional attributes that may benefit plants. The presence of genes associated with salt tolerance and disease resistance suggests a potential role in enhancing plant salt tolerance and managing pathogens. The prevalence of biosynthetic gene clusters is also noteworthy. The analysis of IAA biosynthetic pathways highlights *B*. *swezey*’s potential in recruiting microbial taxa for promoting plant growth (Figures 5, 6). Further plant testing is crucial to confirm these potentials in future research.

Furthermore, microbial taxa’s diverse trophic and metabolic activities underscore their multifaceted roles in oxidative phosphorylation, carbon fixation, and nutrient cycling. This microbial activity significantly improves soil and supports plant development in various ecosystems (Li et al., 2023; Shi et al., 2023). In conclusion, *B*. *swezey* is a key player in shaping a resilient and beneficial rhizosphere microbiome, creating conditions favorable for plant growth. These findings enhance our understanding of *B. swezey’s* complex interactions and offer promise for sustainable agricultural practices in challenging environments.

## 5 Conclusion

This study employed high-throughput sequencing to characterize endophytic bacterial diversity in *A. truncata* (Schrenk) Bunge, and *A*. *eriopoda* (Schrenk) Benth ex Volkens from the Aral Sea region. *A. truncata* exhibited higher richness and diversity. *Pseudomonadota*, *Bacteroidota*, and *Actinomycetota* were common phyla in both halophytes. At the same time, *Bacillota*, *Pseudomonadota*, *Actinomycetota*, and *Bacteroidota* were dominant in *A*. *eriopoda*—Simlilry *Pseudomonadota*, *Actinomycetota*, *Bacteroidota*, and *Chloroflexota* were dominant in *A. truncata*. Biomarkers were used to find changes in the structures of microbial communities. Phylogenetic analysis revealed high diversity, including potential new taxa, and PICRUSt2 highlighted metabolism as a major functional focus. Plant-endophytic bacteria with particular functions are crucial in halophyte adaptability in saline conditions. These findings offer insights into the complex microbial community structure associated with two halophytes from the West Aral Sea’s saline-alkali soil. Future research employing next-generation sequencing (NGS) techniques is essential for investigating the functions of plant-microbe interactions and isolating strains for experimental validation.

Furthermore, this study explored the evolutionary relationships, metabolic capacities, and plant-beneficial potentials of *B*. *swezeyi* strains isolated from halophytes (*A. truncata* and *A*. *eriopoda*). Genomic and physiological analyses revealed their environmental adaptability, diverse carbon source utilization, and high salt-stress tolerance. The enzymatic combination includes proteases, cellulases, and chitinases, highlighting their versatile capabilities. Although genetic variations, comparative genomic analysis showed shared genetic similarities and metabolic features among the strains. These strains displayed significant plant-growth-promoting and antagonistic potentials, suggesting their promising role in addressing global food security challenges. The findings underscore the multifaceted capabilities of *B*. *swezeyi* in fostering plant growth and combating potential phyto pathogens, presenting valuable implications for sustainable agricultural practices and food insecurity.

## Data Availability

The complete raw sequence data related to endophytic bacteria have been deposited in the SRA database of NCBI under accession numbers SAMN17934895 to SAMN17934900. The GenBank/EMBL/DDBJ accession numbers for the draft genomes of *B. swezeyi* are JAYLVX000000000-JAYLWB000000000, respectively.

## Footnotes

^1^https://www.britannica.com/place/Aral-Sea (Accessed July 14, 2024).
