# Supplementary material for: Insight into endophytic microbial diversity in two halophytes and plant beneficial attributes of Bacillus swezeyi
Source: Front Microbiol. 2024 Aug 29;15:1447755. doi: 10.3389/fmicb.2024.1447755 (PMC11391308; doi:10.3389/fmicb.2024.1447755)
Supplement: Supplementary file 1 [file Image_1.pdf]

Insight into Endophytic Microbial Diversity in Two Halophytes and Plant Beneficial Attributes of *Bacillus swezeyi*

Lei Gao[1,3], Li Li[1,2*]

[1] **State Key Laboratory of Desert and Oasis Ecology, Key Laboratory of Ecological Safety and Sustainable Development in Arid Lands, Xinjiang Institute of Ecology and Geography, Chinese Academy of Sciences**

[2] **Xinjiang Key Laboratory of Biodiversity Conservation and Application in Arid Lands, Xinjiang Institute of Ecology and Geography, Chinese Academy of Sciences**

[3] **University of Chinese Academy of Sciences, Beijing 100049, China;**

[*]Authors for correspondence:

Li Li

E-mail: lili.bobo@outlook.com

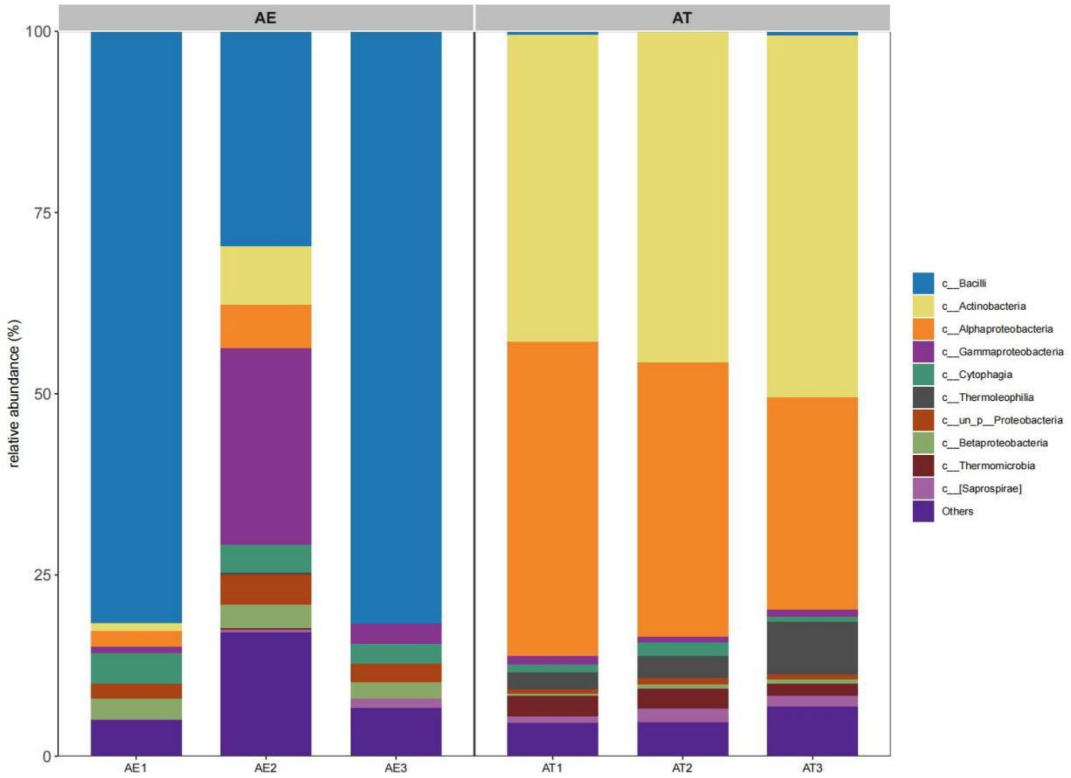

Supplementary Fig. S1 Relative abundances of bacteria at the class level (top 10) in different samples.

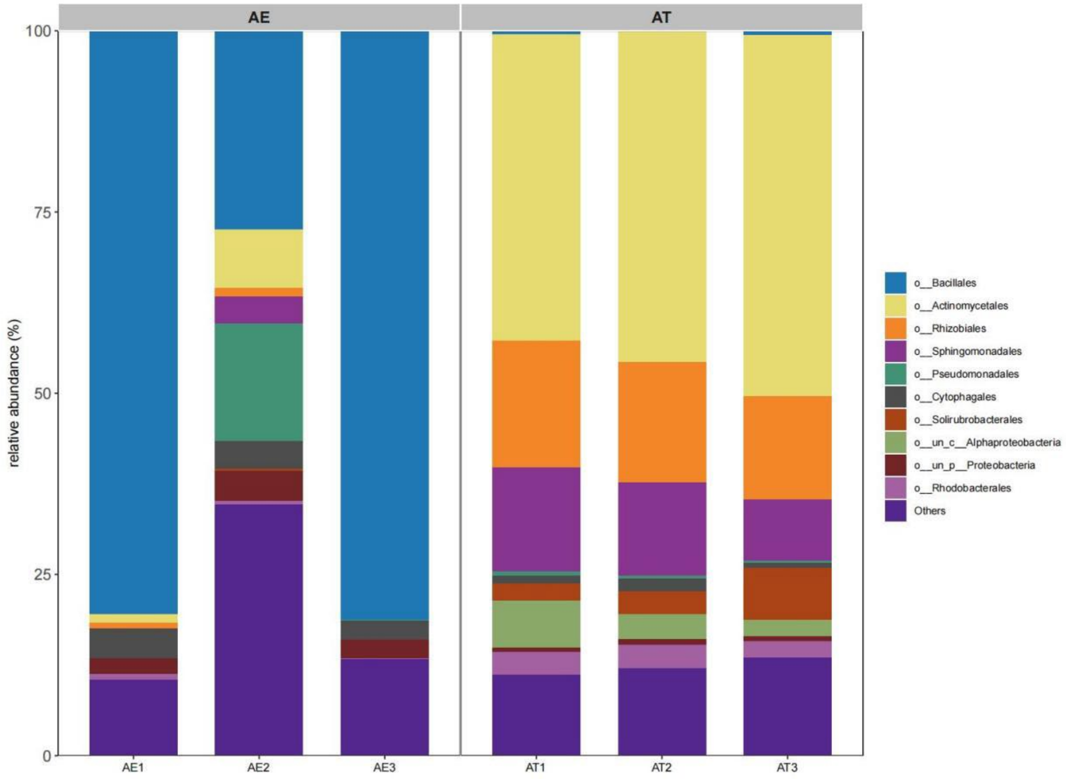

Supplementary Fig. S2 Relative abundances of bacteria at the order level (top 10) in different samples.

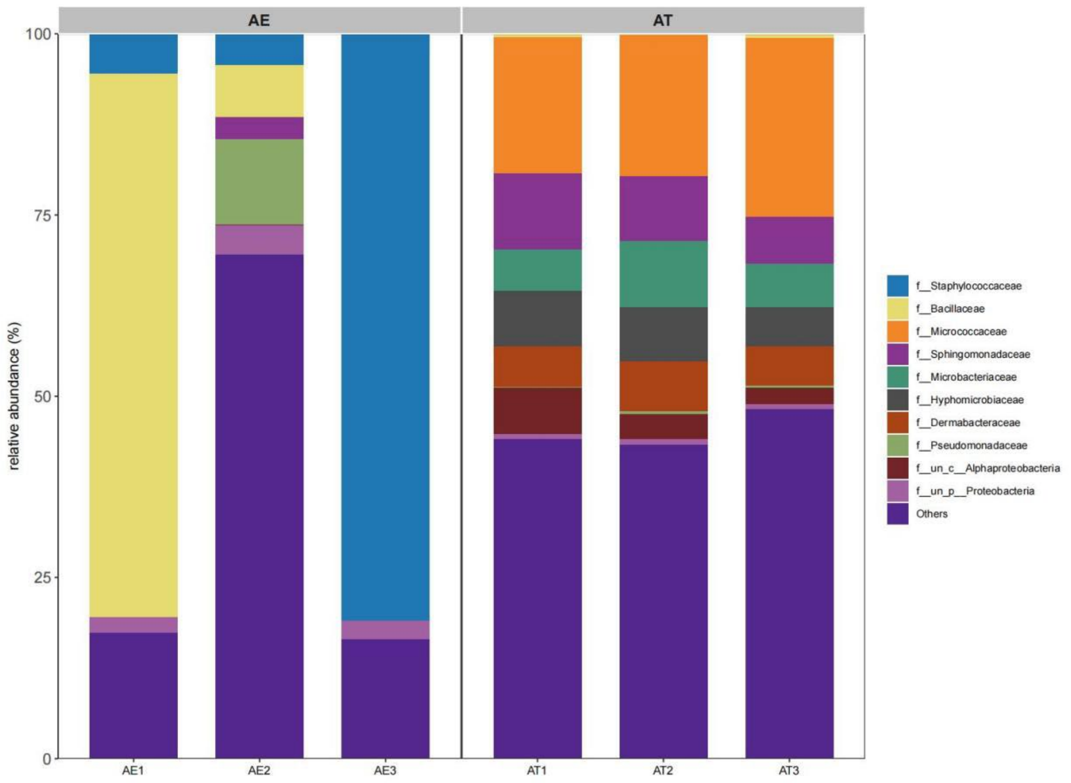

Supplementary Fig. S3 Relative abundances of bacteria at the family level (top 10) in different samples.

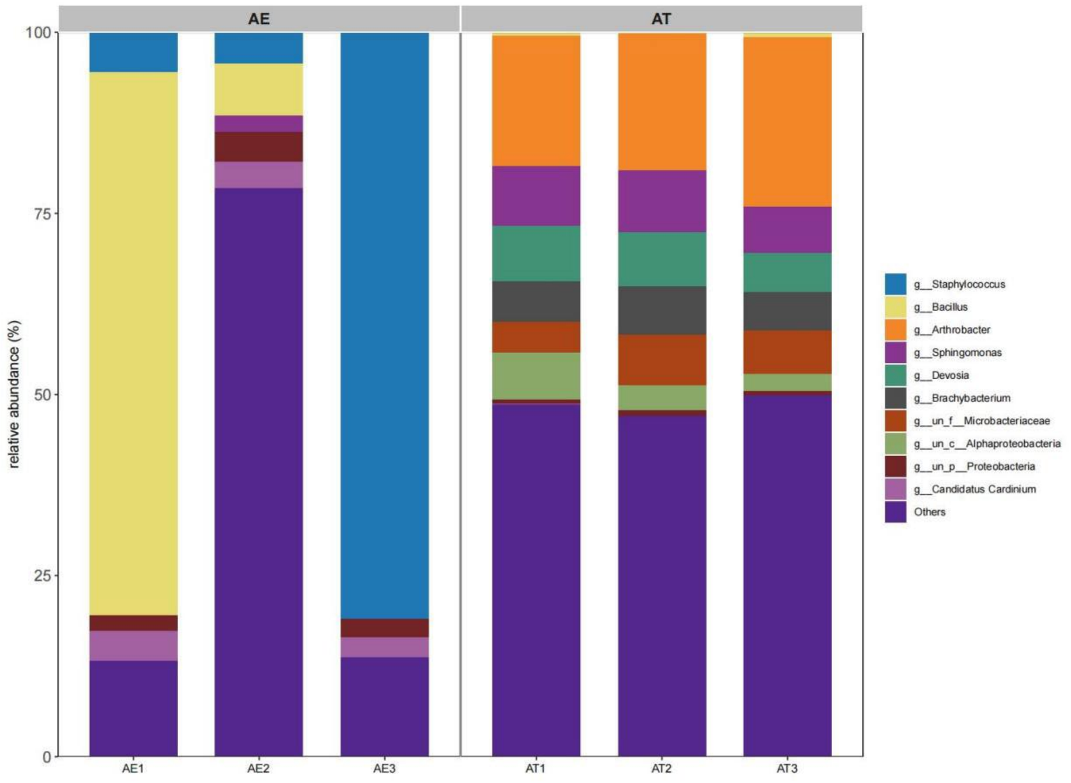

Supplementary Fig. S4 Relative abundances of bacteria at the genus level (top 10) in different samples.
